# Profiles in Criminal Psychopathology: A Multiple Case Report Study of the *p* Factor

**DOI:** 10.3390/ijerph19126960

**Published:** 2022-06-07

**Authors:** Alan J. Drury, Michael J. Elbert, Matt DeLisi

**Affiliations:** 1 United States Probation and Pretrial Services, Southern District of Iowa, Des Moines, IA 50309, USA; alan.drury@iasp.uscourts.gov (A.J.D.); michael.elbert@iasp.uscourts.gov (M.J.E.); 2 Department of Sociology, Iowa State University, 510 Farm House Lane, 203A East Hall, Ames, IA 50011, USA

**Keywords:** psychopathology, *p* factor, crime, criminal offender, correctional client, forensic profile

## Abstract

(1) Background: The one general psychopathology (*p* Factor) theory asserts that a superordinate dimensional construct encompasses underlying forms of psychopathology, but the theory has limited empirical linkages to criminology. (2) Methods: We utilized case reports from 12 male offenders selected from a federal jurisdiction in the central United States who were in the 99th percentile on a composite indicator of psychopathology to advance a qualitative study of the *p* Factor. (3) Results: Clients experienced frequent and often pathological traumatic experiences and exhibited exceedingly early onset of conduct problems usually during the preschool period. Their criminal careers were overwhelmingly versatile and contained numerous offense types, had extensive justice system contacts, and exhibited remarkable deficits in global functioning. Most clients spent the majority of their life in local, state, or federal confinement. Consistent with the theory, clients experienced a generalized psychopathology disposition that had undercurrents of externalizing, internalizing, psychotic, paraphilic, and homicidal features. (4) Conclusions: A qualitative understanding of the *p* Factor and its contribution to offending behaviors among correctional clients complements the statistical approach to developmental psychopathology.

## 1. Introduction

A major conceptual advance in developmental psychopathology, the general psychopathology or *p* Factor asserts that a superordinate dimensional construct known as *p* subsumes all underlying forms of psychopathology (Prior research also examined a unifying psychopathology factor that potentially subsumed other forms of psychiatric diagnosis [1,2,3,4,5,6] before the advent of the *p* Factor. Moreover, [7] theorized that criminal behavior was effectively a clinical disorder with a similar psychopathology approach. Nevertheless, the *p* Factor is a distinct newer theoretical development rooted in this broader tradition). To support their theoretical suppositions, Ref. [8] analyzed eleven forms of psychopathology using data from the Dunedin Multidisciplinary Health and Development Study. These disorders included alcohol dependence, cannabis dependence, dependence on hard drugs, tobacco dependence, conduct disorder, major depressive disorder, generalized anxiety disorder, fear/phobias, obsessive-compulsive disorder, mania, and schizophrenia. Factor analyses indicated these 11 conditions organized into three higher-order factors including an internalizing factor, an externalizing factor, and a thought disorder factor, but their associations with psychiatric outcomes greatly reduced after the extraction of *p*. The *p* Factor had omnibus associations to numerous outcomes including antisocial personality features (e.g., low agreeableness, low conscientiousness, high neuroticism), overall life impairment including suicide attempts, psychiatric hospitalization, welfare usage, and convictions for criminal violence, lower socioeconomic status, greater behavioral disorders, everyday cognitive impairment, and reduced brain integrity and executive functioning.

Additional studies similarly reported evidence of an overarching *p* Factor among diverse samples of data and employing various measurement approaches [9,10,11,12,13,14,15,16,17]. In an overview of their theory, ref. [18], p. 840 advised, “Mental disorders are pervasive in the population; they do not breed true across generations in families; they show little causal specificity; and they do not simply go away, instead often morphing with time into other, different conditions. Indeed, people who experience one condition often experience other co- and future-occurring conditions. These facts possible reflect a quantitatively distributed, stable, generalized liability to develop any and all forms of psychopathology across the life course, which we called *p*”. Although it is a newer theory, the *p* Factor has quickly generated supportive quantitative empirical evidence. 

Despite the visibility of the *p* Factor approach, the perspective has only recently appeared in a criminological context. Using data from 1722 federal correctional clients, the authors of [19] created a 22-item composite indicator of the *p* Factor containing externalizing, internalizing, substance use, paraphilic, and forensic indicators. They found that males, younger clients, and those with greater abuse exposure had higher psychopathology. There was wide variance in psychopathology with nearly 29% of the correctional clients having zero psychopathology. About 60% of the sample had average to very high psychopathology. In subsequent multivariate regression models, the *p* Factor was strongly and positively associated with total, violent, sexual, property, weapon, and drug arrest charges net the effects of sex, race, age, and seven traumatic experiences. The size of these effects was substantial with z-scores ranging from 4 to over 20. Their findings suggest that the *p* Factor is a robust risk factor for multitudinous forms of antisocial behavior. 

Although the *p* Factor spurred research surrounding the conceptualization and measurement of global psychopathology, its salience to criminology is limited and there are several research gaps. For instance, although the *p* Factor is highly heritable (To illustrate, the authors [9] analyzed twin data from the Twins Early Development Study and reported that 50% to 60% of variance in the *p* Factor is attributable to genetic factors and between 49% to 78% of the shared variance in continuity of psychopathology across childhood and adolescence was also owed to genetic factors), the environment also plays a critical role in its development, thus a focus on the rearing environments of criminal offenders, where disparate forms of abuse and neglect are commonplace [20,21,22,23] is needed. The original *p* Factor study eschewed low prevalence phenomena that although rare in the general population, are much more prevalent among the correctional population. Thus, the degree to which forensic indicators, such as homicidal ideation, paraphilic disorders, such as pedophilia and sexual sadism, and severe behavioral disorders, such as Intermittent Explosive Disorder and Antisocial Personality Disorder are constitutive of a general psychopathology factor is lacking. Finally, extant research on *p* is quantitative and generally involves exploratory factor analysis, confirmatory factor analysis, and structural equation modeling. Unfortunately, analytical sophistication does not address the intricate details of an offender’s upbringing, their exposure and reactions to abuse and neglect and their subsequent adaptation to institutional placements, and how their criminal activity waxes and wanes as a function of their underlying psychopathology. In short, we know very little about what offenders with high *p* Factor scores are like. We know very little about their life experiences. A qualitative understanding of the *p* Factor and its contribution to offending behaviors among correctional clients is missing. The current study sought to fill this void in the literature.

## 2. Materials and Methods

The current study employed a multiple case report approach. Common in psychiatry and the forensic sciences, the case report is a qualitative method based on exhaustive access to information to describe symptoms or behaviors that are unique or pathological in their onset, presentation, severity, and developmental course. Case reports are rare in criminology, but are somewhat common in studies of multiple homicide offenders [24,25,26]. We utilize case reports from 12 male offenders selected from a federal jurisdiction in the central United States who were in the 99th percentile on a composite indicator of psychopathology. The composite indicator of additive index included lifetime diagnoses/evidence for Oppositional Defiant Disorder, Conduct Disorder, ADHD, Antisocial Personality Disorder, Other Personality Disorder, PTSD, Intermittent Explosive Disorder, Generalized Anxiety Disorder, Major Depressive Disorder, Dysthymia, Alcohol Dependence, Cannabis Dependence, Opioid Dependence, Cocaine Dependence, Methamphetamine Dependence, Self-Harm/Mutilation, Suicidal Ideation, Suicide Attempts, Homicidal Ideation, Pedophilia, Sexual Sadism, and other paraphilic disorder. All of these correctional clients served a confinement sentence in the Bureau of Prisons and are currently on supervised release in the community. The case reports rely on archival records including official and self-reported criminal history, presentence investigation reports, state and federal correctional records, psychological reports, case history information, archival supervision data, and collateral legal documents. The Chief District Judge in this federal jurisdiction provided research approval for the study and all clients voluntarily signed an informed consent form granting use of anonymized information. The study has exempt university IRB status since it relies on archival records and did not involve human subjects.

## 3. Results

### 3.1. Mr. A

Mr. A is a 42-year-old white male on supervised release for escape from a federal residential reentry center. Beginning with his first arrest at age 11, Mr. A has a lengthy criminal history with 68 arrest charges primarily for violent offenses including assault, robbery, intimidation, child abuse, and domestic violence, and property crimes including burglary, forgery, and theft. Although he is Caucasian, Mr. A was involved with the African American Bloods street gang during adolescence and early adulthood. 

Mr. A’s early life was tumultuous and involved numerous traumatic experiences. His parents were criminal offenders, had multiple substance use and other psychiatric disorders, and sold drugs out of their family home. He incurred chronic and severe physical abuse, emotional abuse, physical neglect, emotional neglect, educational neglect, and medical neglect. During periods of methamphetamine intoxication, his parents would beat Mr. A with tree branches, pieces of wood, extension cords, and other household objects and choke him until he lost consciousness. To flee the abuse, Mr. A eloped from home at age 14 and was intermittently transient or placed in juvenile justice custody. 

These traumatic experiences presaged coextensive psychopathology. In terms of lifetime diagnostic history, clinicians diagnosed Mr. A with ADHD, Intermittent Explosive Disorder, Bipolar I Disorder, PTSD, Generalized Anxiety Disorder, Methamphetamine Dependence, Cannabis Dependence, Major Depressive Disorder, and Cocaine Abuse. During adulthood, Mr. A self-reported addiction to synthetic marijuana and that he experienced psychosis, seizures, and violent outbursts when using the drug. Mr. A has chronic suicidal ideation, multiple suicide attempts, and acknowledges that his drug history was a combination of suicide attempts and enjoyment of the substances. 

Mr. A has lifelong maladjustment with the criminal justice system. During his most recent confinement in the Bureau of Prisons, he engaged in repeated misconduct including five violent assaults. Most of his probation, parole, and supervised release sentences resulted in revocation due to complete refusal to comply with conditions. For instance, on one sentence, Mr. A committed new crimes, failed to provide proof of employment or submit employment reports, failed to pay child support, used drugs, associated with known offenders, failed to report for drug testing, and consistently missed appointments with his probation officer. Mr. A completed his general educational development (GED) certificate while in federal prison, is chronically unemployed, and has historically sustained himself economically through petty thefts, public assistance, and reliance on family or girlfriends.

### 3.2. Mr. B

Mr. B is a 39-year-old black male on supervised release for attempted interference with commerce by robbery. He has a 2-state criminal history totaling 78 career arrest charges including 29 police contacts as a juvenile with his first police contact at age 11. Although his offending history is diverse in terms of violent, property, drug, weapon, and public order crimes, Mr. B’s offending history is particularly violent in nature and includes 27 arrest charges for violent offenses including murder, attempted murder, armed home invasion, armed robbery, assault while participating in a felony, domestic violence, battery, and disorderly conduct. He recurrently used weapons during violent assaults and would engage in violent acts across contexts (e.g., in school settings, in family and domestic contexts, or while in custody). Mr. B has eight separate commitments to juvenile confinement facilities, state prison, and federal prison. Whether in confinement or the community, Mr. B virtually never complied with court orders and engaged in institutional misconduct and repeated violations of supervision including use of drugs, failure to comply with treatment, failure to pay fines and restitution, failure to secure employment, and escape. 

Mr. B’s parents never married and his rearing environment was void of abuse. He lived in a poor neighborhood where crime and violence were common and joined the Gangster Disciples street gang at age 11. Subsequently, he had multiple out of home placements for conduct problems. He was expelled from school at age 15 for fighting and ultimately earned his high school diploma while incarcerated. He has limited work experience as a laborer and historically performs odd jobs for family members and collects public assistance. 

Consistent with his offense history, Mr. B’s psychiatric history involves mostly externalizing conditions. He has lifetime diagnoses for Conduct Disorder, Antisocial Personality Disorder, Intermittent Explosive Disorder, Narcissistic Personality Disorder, ADHD, Cannabis Dependence, Alcohol Dependence, Cocaine Abuse, and anxiety/depression. He has described pervasive thoughts about hurting other people regardless if they bothered him in any way and that he often acts on these impulses with no regrets. On nearly ten occasions, Mr. B uttered homicidal statements during the course of perpetrating an assault, stabbing, or shooting.

### 3.3. Mr. C

Mr. C is a 39-year-old white male on supervised release for failure to register as a sex offender or SORNA. Mr. C has alarming behavioral history of sexually inappropriate and violent conduct including raping two of his sisters at age 14 for which he received probation, later revoked, and ultimately placed in juvenile confinement. As an adult, Mr. C has convictions for child rape, communicating with a minor for immoral purposes, and four instances of failure to register as a sexual offender all of which resulted in imprisonment. A versatile offender, Mr. C has a 6-state criminal history including 30 total arrest charges for additional crimes spanning violent, property, drug, and public-order offenses. 

Mr. C’s maladjustment emerged early in life with onset of conduct problems and externalizing features at the age of nine with his first police contact at age 11 years. Early conduct problems were primarily of a sexual nature including masturbating in front of other children and attempting to coerce other children into sexual activity. His rearing environment involved multiple adverse experiences including poverty, emotional and physical abuse from his father, and sexual victimization by an older teenager when his family resided at a transient hotel. Mr. C’s biological parents had substance use disorders. 

He has comorbid substance use and other psychiatric history including lifetime diagnoses for Major Depressive Disorder, Methamphetamine Dependence, and Cocaine Dependence. He experienced acute psychosis especially when abusing methamphetamine, chronic suicidal ideation since childhood, and attempted suicide on multiple occasions during adolescence and adulthood. He acknowledges that much of his substance abuse was “passive aggressive” suicide attempts and his drug history includes alcohol, marijuana, methamphetamine, cocaine, cocaine base, heroin, LSD, MDMA, GHB, designer drugs, and benzodiazepine. 

His criminal career is remarkable for consistent refusal to comply with court orders and supervision conditions. Mr. C traveled the country itinerantly and consistently failed to appear in court, usually declined to complete registration requirements despite his sexual conviction history, absconded from probation and parole, and consistently drove a vehicle without license or insurance. Mr. C prefers to live “off the grid” and is chronically transient. He completed a general educational development (GED) certificate during adolescent confinement and has limited employment history as a laborer or street performer.

### 3.4. Mr. D

Mr. D. is a 33-year-old white male on supervised release for coercion and enticement. He has a relatively limited official criminal history with 11 total arrest charges for violent, sexual, and alcohol-related offenses and his only confinement was for his instant offense. He graduated high school, consistently maintained employment in retail and service industries, and served in the military. Relative to other criminal offenders in the federal system, his socioeconomic functioning is high.

From age two months, Mr. D incurred significant physical abuse from his father resulting in multiple broken bones, pervasive contusions and abrasions, and several events that produced loss of consciousness that required resuscitation. His parents divorced when Mr. D was a toddler and although the paternal abuse ended, his mother neglected him throughout his childhood. Several babysitters and boyfriends of his mother sexually abused Mr. D until approximately five years old and he experienced recurrent physical abuse, emotional abuse, and emotional neglect throughout adolescence. 

Mr. D first displayed conduct problems at the age of four in which he would go into uncontrollable rages and become highly aggressive. He reported experiencing mood swings, racing thoughts, visual and auditory hallucination, and pronounced anger that occasionally resulted in “blacking out” and memory loss. During these anger experiences, Mr. D reported homicidal thoughts. In later childhood, he set fires, engaged in promiscuous sexual behavior with children, adults, and animals, and substance use. 

Mr. D experiences long-term neurological and neuropsychological problems relating to the abuse incurred during infancy and has an extraordinary range of mental health problems. His lifetime diagnostic history includes ADHD, Oppositional Defiant Disorder, Adjustment Disorder with Depressed Mood, PTSD, Personality Disorder NOS, Borderline Personality Disorder, Antisocial and Dependent Personality Disorder traits, Generalized Anxiety Disorder, Depressive Disorder NOS, Alcohol Abuse, and Major Depressive Disorder. Mr. D has lifelong suicidal ideation and attempted suicide on at least ten occasions and incurred significant injuries from some of these attempts. He engages in self-injurious and self-mutilating behaviors and cuts himself to, according to Mr. D, relieve pressure and experience pain. Mr. D reported homicidal ideation at various stages of his life and paraphilic disorders including symptoms of sexual sadism, pedophilia, exhibitionism, bestiality, pornography addiction, and has admitted to raping intimate partners.

### 3.5. Mr. E

Mr. E is a 55-year-old black male on supervised release for armed bank robbery. He has a violent, voluminous 2-state criminal history with 63 total arrest charges, 22 convictions, and 10 separate prison commitments. Although Mr. E is a versatile offender with multiple offense types on his record, he has 20 arrest charges for weapons offenses and 19 arrest charges for violent crimes. Mr. E has been arrested for first-degree murder on three separate occasions, but was never prosecuted for those charges. He has 10 separate armed robbery arrests or convictions, the earliest occurring when he was age 15 years. 

His rearing environment was void of traumatic experiences until his father died when Mr. E was age 10 years, after which Mr. E joined the Gangster Disciples gang and began exhibiting significant conduct problems including nine police contacts and a commitment to a juvenile reformatory. Mr. E’s mother has multiple psychiatric problems and two of his brothers also have extended arrest activity and incarceration history. 

Mr. E suffers from a range of health problems and has been shot on four separate occasions, some of these injuries produced lifelong physical impairments. His lifetime diagnoses include Schizoaffective Disorder, Major Depressive Disorder, Schizophrenia, and Borderline Intellectual Functioning. He has experienced command auditory hallucinations to harm himself and has pervasive suicidal ideation and multiple suicide attempts especially during periods of incarceration. Beginning at age 13, Mr. E has intermittently abused marijuana, cocaine, alcohol, heroin, cocaine base, PCP, and prescription opiates and been dependent on most of these substances. Mr. E was expelled from high school for fighting and has never worked. He historically sustained himself by selling drugs and committing robberies and property crimes.

### 3.6. Mr. F

Mr. F is a 40-year-old American Indian male on supervised release for possession of unregistered firearms. With an antisocial conduct onset at age 7 years, Mr. F accumulated a 3-state criminal history with 143 total arrest charges including 41 violent arrest charges and three prison commitments. His offense history includes multiples rapes, assaults with intent to murder, and numerous aggravated assaults with weapons. He exhibited pervasive homicidal ideation throughout life and made homicidal utterances during the course of many of his violent attacks. 

Mr. F’s rearing environment was highly dysfunctional as both parents and an older sibling were active criminal offenders who abused multiple drugs. He incurred a litany of trauma experiences spanning sexual abuse, physical abuse, emotional abuse, emotional neglect, medical neglect, physical neglect, and frequent exposure to criminal activity and substance use. He was occasionally placed into locked closets or car trunks so his parents would not have to monitor him. Mr. F had 13 police contacts as a juvenile and was recurrently placed in juvenile detention and other facilities for significant conduct problems. 

Mr. F struggled with acute suicidal ideation and multiple suicide attempts beginning at the age of seven. His expansive diagnostic history includes Major Depressive Disorder, PTSD, Anxiety Disorder NOS, Alcohol Dependence, Cocaine Dependence, Amphetamine Dependence, Cannabis Dependence, Personality Disorder NOS, Dysthymic Disorder, Acute Stress Disorder, Intermittent Explosive Disorder, Avoidant Personality Disorder, Mixed Personality Disorder with Schizoid and Narcissistic traits, and Opiate Dependence. Although frequently suspended for behavioral problems and fights, Mr. F nevertheless graduated high school, served in the military, and maintained intermittent employment throughout adulthood.

### 3.7. Mr. G

Mr. G is a 37-year-old white male on supervised release for possession of child pornography. His only prior criminal history involved a burglary and sexual assault of a 7-year-old child when he was 16 years of age. Authorities removed Mr. G from his birth parents’ custody during infancy due to severe physical abuse (both biological parents were active offenders and drug users) and he was subsequently adopted. Mr. G suffered from sexual abuse from ages 6 to 16 years that was perpetrated by multiple adult males. 

Despite his limited official criminal history, Mr. G evinced versatile and severe conduct problems beginning at the age of five. These acts include fire setting, attempted sexual assault, assault, cruelty to animals, property damage, sexually inappropriate behavior (e.g., exhibitionism and voyeurism), aggression, and noncompliant and uncooperative conduct with adults. He has substantial executive dysfunction stemming from prenatal drug and alcohol exposure and severe psychopathology involving psychosis, suicidal thoughts and behaviors, rape fantasies, and homicidal ideation. At multiple periods during adolescence, Mr. G stated that auditory command hallucinations urged him to murder family pets, family members, and other community members although he never acted on these impulses. He experiences similarly violent fantasies pertaining to the molestation of children. 

His lifetime diagnoses include ADHD, Impulse Control Disorder, Major Depressive Disorder, Conduct Disorder, Borderline Personality Disorder, Oppositional Defiant Disorder, Borderline Intellectual Functioning, Fetal Alcohol Effects, Generalized Anxiety Disorder, Dysthymic Disorder, Pedophilia, Sexual Dysfunction NOS, Schizoid Personality Disorder, Avoidant Personality Disorder, Dependent Personality Disorder traits, and Bipolar Disorder with Psychotic Features. He experimented with alcohol once during early adulthood and otherwise has never used illicit drugs. Mr. G graduated high school, receives Social Security disability benefits, and has consistently maintained employment. 

### 3.8. Mr. H

Mr. H is a 28-year-old black male on supervised release for escape. He has a violent and versatile 2-state criminal history including 30 arrest charges with 21 police contacts as a juvenile beginning at age 7 years. Mr. H’s offense history spans violent crimes (battery, assault, armed robbery), sexual crimes (rape, SORNA), property crimes (burglary, theft, arson), drug crimes (sale of cocaine and marijuana), and numerous public order offenses. He exhibited unrelenting noncompliance with jail, probation, state prison, and federal prison sentences including multiple violations, revocations, institutional misconduct, absconding, and escape. 

Mr. H’s parents never married, frequently beat him, and lost custody of Mr. H when he was age 10 years when he subsequently joined the Black Stones street gang. Due to his chronic delinquency, Mr. H was placed in detention, foster homes, and confinement facilities. Amazingly, his onset of antisocial conduct occurred at the age of two when he had police contact after leaving his home and playing with gasoline and matches at a nearby park.

He experienced lifelong suicidal ideation and attempted suicide several times during childhood and adolescence. Mr. H also has evidence of self-injurious behaviors, dissociative experiences, and thought disturbances. His lifetime diagnoses include ADHD, Pervasive Developmental Disorder, Major Depressive Disorder, Bipolar I Disorder, Oppositional Defiant Disorder, Schizoaffective Disorder, Cannabis Dependence, and Antisocial Personality Disorder. 

### 3.9. Mr. I

Mr. I is a 33-year-old white male on supervised release for distribution of child pornography. Beginning with his first police contact at age 14 years, Mr. I has a 2-state criminal history with 25 total arrest charges of which 14 are for sexual offenses. Six of these crimes include rape, gross sexual imposition, and lewd and lascivious acts, all of which involved children as victims and two of the victims were his younger sisters.

His rearing environment was adverse as both parents exhibited multiple mental health problems and Mr. I was frequently placed in foster care. At several points during his childhood, Mr. I was sexually abused by adults and adolescents while in foster care and by various babysitters and neighbors. After an incident at the age of nine where Mr. I attempted to stab his brother with a knife, he began psychiatric care. His lifetime diagnoses include ADHD, Bipolar II Disorder, PTSD, Methamphetamine Dependence, and Cannabis Dependence. Mr. I has pervasive suicidal ideation and multiple suicide attempts, graduated high school, and has sporadic employment history.

### 3.10. Mr. J

Mr. J is a 33-year-old black male on supervised release for mailing threatening communication. He has a 3-state criminal history with 15 total arrest charges with 11 offenses for violent and sexual crimes, and his first arrest occurred at age 12 years. His conviction history includes threats against the President of the United States, rape, criminal sexual abuse, intimidation, aggravated assault, assault, burglary, and fugitive from justice.

Mr. J was born to adolescent parents who abandoned him during infancy, and during early childhood, he experienced multiple forms of abuse and neglect. After a brief period of rearing from his grandparents, Mr. J became a ward of the state at the age of five. At that age, he began to exhibit increasingly unmanageable behavioral problems including sexual aggression, fire setting, threatening to kill teachers, foster parents, and other children, assaults of teachers and other children in foster care or school, urinating in his classroom, enuresis, defiance, weapons use, and other delinquent acts. He was recurrently placed in therapeutic foster care homes, detention centers, and juvenile training schools. 

Mr. J has expansive psychopathology. His lifetime diagnoses include ADHD, Oppositional Defiant Disorder, Pervasive Developmental Disorder, PTSD, Impulse Control Disorder, Major Depressive Disorder, Reactive Attachment Disorder, Bipolar I Disorder, Dysomnia, Personality Disorder NOS, Alcohol Dependence, and Antisocial Personality Disorder. Aside from alcohol, Mr. J has never tried an illicit substance and refuses any medications prescribed to treat his psychiatric conditions. He never completed high school, does not work, and sustains himself on Supplemental Security Income when in the community. Otherwise, he has been incarcerated for most of his life. 

Throughout his life, Mr. J experienced poorly modulated homicidal ideation directed at friends, family, and public figures/political leaders. In multiple contexts, his conduct is highly erratic, impulsive, and violent. He exhibited significant maladjustment during his six commitments to confinement facilities and engaged in high levels of institutional misconduct. During his most recent Bureau of Prison imprisonment, Mr. J had 37 violations including nine for violent assaults against staff and other prisoners. 

### 3.11. Mr. K

Mr. K is a 47-year-old black male on supervised release for threat against the President of the United States. He has a 4-state criminal history with 29 arrest charges of which 24 are for violent and sexual offenses including sexual assault of a child, assault, willful injury, rape, assault on police officers, indecency with a child, and inmate assault with bodily fluids. During his five incarcerations, Mr. K engaged in voluminous institutional misconduct including staff assaults, throwing bodily fluids and feces at correctional staff, fire setting, racial threats, suicide attempts, and dozens of other rule violations. His sexual victims span children to middle-aged adults, and Mr. K has an active detainer for state civil commitment proceedings as a sexually violent predator when his federal supervision is complete.

His rearing environment was mostly unremarkable for traumatic experiences with the exception of sexual abuse occurring at the age of four. Mr. K has myriad mental health problems and lifetime diagnoses for Schizoaffective Disorder, Bipolar I Disorder, Borderline Personality Disorder, Antisocial Personality Disorder, Pedophilia, Personality Disorder NOS, PTSD, and Malingered psychosis. He has lifelong suicidal and homicidal ideation and numerous suicide attempts occurring since childhood. 

Uniquely, Mr. K engages in extraordinary self-mutilation that primarily involves swallowing non-food objects (pica) and placing various objects, such as pencils, staples, pens, toothbrushes, buttons, and metal pieces into his urethra. He has never used drugs or alcohol. Mr. K graduated high school and is chronically unemployed with occasional work as a day laborer or fast-food employee.

### 3.12. Mr. L

Mr. L is a 53-year-old white male on supervised release for felon in possession of body armor. He has a voluminous single state criminal history with 79 arrest charges for versatile crimes but primarily violent (aggravated assault, domestic abuse, assault, interference with official acts causing injury), weapon (assault with deadly weapon, possession of a firearm by felon, threaten with weapon), and property (burglary, auto theft, larceny) offenses that resulted in 12 separate prison commitments. Mr. L is particularly recalcitrant and violent with police and incurred additional charges on 13 of these arrest occasions. 

Mr. L’s rearing environment was void of abuse, but his parents divorced when he was five years old, at which he was already displaying significant conduct problems and behavioral impairment. He was frequently placed in group homes and juvenile justice settings for his conduct problems and unmanageable behavior. He was hospitalized numerous times during adolescence and adulthood for suicidal and homicidal ideation and suicide attempts. During these times, he reported auditory command hallucinations to kill himself or others. 

His lifetime diagnoses include Major Depressive Disorder, Antisocial Personality Disorder, Intermittent Explosive Disorder, Depressive Disorder NOS, Cannabis Dependence, Alcohol Dependence, Methamphetamine Dependence, Adjustment Disorder with Mixed Features of Mood and Conduct, and Borderline Intellectual Functioning. He was expelled in fourth grade for fighting and has not completed any educational degree or program, receives Social Security Disability benefits, and has sporadic work history as a laborer. Mr. L has spent the majority of his life in local, state, or federal custody. Table 1 contains summary profiles for all cases. 

## 4. Discussion

Research development on the *p* Factor is highly quantitative in its approach, which obscures the daily challenges that clients with very high psychopathology experience, and how those challenges complicate their behavioral functioning. Drawing on 12 case reports from federal correctional clients on supervised release, we provide qualitative data that reveal the extraordinary range of conduct problems and psychiatric morbidity clients with high *p* exhibit. Several important themes emerged. 

Consistent with the *p* Factor theory [8] and earlier criminological conceptualizations [7], the case reports reveal the existence of a general psychopathological impairment that contained elements of externalizing, internalizing, and psychotic features although the specific blend of conditions varied to a certain degree. Generally, their psychopathology had an early onset that frequently predated adolescence and their developmental course supported both heterotypic and homotypic continuity through adulthood, which is consistent with other research [27,28,29]. Given many of the conditions in these case profiles have moderate to high heritability, it is unsurprising that several of the offenders exhibited clinical behavioral impairments even before entering school and had early justice system intervention. Indeed, by the age of ten, several of these clients already had been in custody for multiple years.

Broad considerations of psychopathology, such as within the *p* Factor theoretical context shed light on the multifactorial causal structure of serious antisocial behavior. A host of genetic, neurological, and environmental inputs contribute to the antisocial development seen among these correctional clients. In some cases, such as where there was significant prenatal exposure to drugs and alcohol, the neurological impairments are evident earlier in life, and these impairments fostered developmental problems in elementary school. There is ample evidence of the intergenerational transmission of abuse, antisocial conduct, and psychiatric conditions, and the case profiles exemplified those processes. Although some offenders certainly had prosocial parents and rearing environments that were unremarkable for abuse, most did not. Instead, the typical profile involved one or more parents, siblings, or other family members who were actively involved in crime, actively using drugs or selling drugs in the family home, and whose psychopathology and conduct problems contributed to the high prevalence of abuse and neglect seen therein. Consistent with this line of research, several of the current clients replicated these behaviors given the frequency with which they were arrested for domestic violence, child abuse, and drug crimes, and that several of them lost their parental rights due to abuse and neglect in the home. 

Criminal psychopathology among these clients had catastrophic effects on their global functioning. Most failed to complete high school often for conduct-related expulsion, had little to no legitimate employment history, and survived on public assistance. It was common for offenders to rely on family members or intimate partners for sustenance or survive via the proceeds of criminal activity. These socioeconomic problems pale to the sheer quotidian struggles that some of these clients face in terms of their basic cognitive, interpersonal, and emotional functioning. Whether it is psychosis, polysubstance dependence, suicidality, paraphilic conduct, or some combination of these challenges, these clients face extreme difficulties. These impairments put into context the various challenges that serious offenders have when attempting to reenter society after confinement [30,31,32,33], and also offer insight why so many chronic offenders continually fail on community supervision.

Although recidivism data among offenders are generally grim, and the majority of the current clients were habitual offenders with low functioning, it is also important to note that three of the 12 clients, 25%, had moderate to high global functioning. Despite chronic and severe exposure to multiple forms of abuse and neglect, these offenders did not just eke out a basic existence, but instead managed to consistently remain employed and maintain a residence. The notion of serious offenders with extensive trauma exposure partially overcoming those experiences is consistent with the concept of an orchid effect in developmental psychopathology. It also showcases the value of qualitative exploration of data even at empirical thresholds (e.g., 99th percentile on the *p* Factor) where one would not expect evidence of normative behavioral functioning. 

Trauma experiences are intimately connected with conduct problems and emotional problems, and likely in multifaceted ways. In some cases, there appears to be a direct causal pathway between adverse experiences and subsequent maladjustment as seen in several profiles where the conduct is commensurate with the abuse incurred, for instance, sexual abuse leading to sexual offending or physical abuse leading to violent offending [34,35,36,37]. In other cases where conduct problems emerged extremely early, such as from the age of two to four, there was likely evocative effects where externalizing problems increased the likelihood of negative parenting and abuse, which in turn exacerbated the child’s conduct problems. Trauma exposure was also seen in multiple contexts in most of these client’s rearing environment where parental abuse and neglect resulted in out of home placements that also frequently included additional abuse exposure. As a result, many clients eloped from foster and group home settings only to be subsequently placed in detention settings, many of which had their own culture of violence. 

Our qualitative data also shed light on the truly horrific conditions under which some correctional clients lived during their formative years. As shown in the profiles for Mr. A, Mr. D, Mr. F, Mr. G, Mr. I, and Mr. J, in particular, some were born to drug-addicted parents and have lifelong neurological problems attendant to that abuse, and others incurred severe abuse during infancy and were removed from the home. The abuse and neglect were a constant feature of their childhood. A bevy of quantitative studies indicates that trauma/averse childhood experiences are strongly associated with serious and chronic offending [38,39,40,41,42,43,44]. Our data reveal how trauma experiences likely engender a consuming psychopathology, often with homicidal and suicidal overtones, that facilitates substance use and crime. Reading the life histories of these offenders is a distressing and truly depressing experience. In sum, pathological abuse and neglect is a robust environmental feature of antisocial development, and a tragic one [40,45].

The *p* Factor theory presents as a general theory, and as such, has interesting overlap with other general theoretical concepts in criminology namely self-control [46,47] and psychopathy [48,49,50,51] (Comparative studies of the *p* Factor and self-control would be interesting because Caspi, Moffitt, and colleagues developed and advocate both general conceptual models for psychopathology and antisocial behavior [47,52,53,54,55,56]. An exciting research opportunity relates to the convergent and divergent validity between *p* and self-control). Several diagnostic conditions especially cluster B personality disorders evince a set of traits that are compatible with Gottfredson and Hirschi’s low self-control profile one that has considerable empirical linkages to conduct problems [57,58]. Indeed, a basic deficit, refusal, or unwillingness to regulate one’s conduct appears in all of our data and is most apparent in the noncompliance with judicial orders and correctional sentences. Unfortunately, the current data does not include a self-control measure, but a promising avenue for future research is to see whether self-control measures can withstand controls for other forensic constructs including Antisocial Personality Disorder, Intermittent Explosive Disorder, and homicidal ideation that also potentiate self-control problems [59,60]. 

There were limited psychopathy data in these clients’ correctional files (e.g., PCL-R scores), but review of their criminal history and other file information clearly shows that psychopathy is often present. By our calculations, Mr. A, Mr. F, Mr. G, Mr. I, Mr. J, and Mr. K would score in the moderate range on the PCL-R (total scores >20 but <30) and Mr. B, Mr. C, Mr. D, Mr. E, Mr. H, and Mr. L would score in the clinical range on the PCL-R (total scores ≥30). Put another way, half of these case profiles of clients with exceedingly high psychopathology are also psychopaths. Overwhelmingly, their psychopathic features are concentrated in the affective, lifestyle, and antisocial domains with less evidence of interpersonal features. We suspect that some of the other conditions inherent to the *p* Factor including educational deficits, cognitive problems, brain insults, and reduced global functioning are so debilitating that they undermine the potential for interpersonal motivation and conversational acumen. 

In terms of the offending careers of these clients, there was overwhelming evidence of criminal versatility spanning a range of offense types. This is consistent with most prior research of criminal careers [61,62,63,64]. Moreover, clients with more worst substance abuse histories frequently had the lengthiest arrest histories that is consistent with the “worst of both worlds” thesis [65,66]. The case profiles also revealed the idiosyncratic nature of offending and substance use where there also appears to be evidence of criminal specialization or least preferences for certain types of crime. For instance, most offenders had extraordinarily severe substance use histories involving dependence on numerous substances whereas other offenders, such as Mr. G and Mr. J never tried any illicit drugs and only had brief, normative experimentation with alcohol. Mr. K *never* tried drugs or alcohol yet engaged in the most pervasive and self-destructive personal harm behaviors. In terms of specialization, Mr. E was a very active armed offender who committed at least ten armed robberies and accrued three arrests for first-degree murder, and Mr. L had a similar proclivity for armed violence. Studies of offender specialization and versatility are highly quantitative and mechanistic in their approach, and considering the broader psychopathological features that an offender has can shed light on why an individual might gravitate toward, and even express a preference for, a specific form of antisocial conduct.

We acknowledge the generalizability limitations associated with a case report approach using 12 male offenders with high psychopathology and encourage additional qualitative inquiries about the *p* Factor among justice system involved women and other offender typologies (e.g., homicide offenders, sexual offenders, life-course-persistent offenders). This approach adds data nuance, clinical and forensic insights, and an empathic scientific inquiry into the protean psychopathology that typifies those involved in the criminal justice system.

## 5. Conclusions

In the setup for the present study, we observed that statistical sophistication does not address the intricate details of an offender’s upbringing, their exposure and reactions to abuse and neglect and their subsequent adaptation to institutional placements, and how their criminal activity waxes and wanes as a function of their underlying psychopathology. We observed that extant research reveals very little about what offenders with high *p* Factor scores are like, and how their lives unfolded. In our practitioner roles, we have heard countless offenders indicate that due to the various negative circumstances of their life including their neglectful and abusive parents, the depriving neighborhoods in which they lived populated by a litany of negative influences, and the profound risk factors that they themselves exhibit, that they “never had a chance”. Moreover, since they feel they never had a chance, many develop a highly self-destructive, dysphoric, intentionally antisocial behavioral style that wreaks havoc on their school career, family life, and work life, and constitutes what is often lifelong engagement with the justice system and diverse forms of correctional supervision. Even there, many offenders are thoroughly recalcitrant and continue to behave in aggressive and noncompliant ways. Keeping with the never had a chance ethos, most will never disengage from correctional supervision. The real and perceived institutional hardships of jail, prison, and community supervision at midlife have replaced the institutional interventions that occurred in their first decades of life. Contributing to and resulting from these life events is a formidable psychopathological profile that drives criminal behavior. 

## Figures and Tables

**Table 1:** Case report profiles.

Client	Offense	Criminal Career	Justice System Involvement	Abuse Exposure	Neglect Exposure	Lifetime Diagnostic History	Substance Use Profile	Remarkable Features
A	escape	childhood-onset, life-course-persistent, versatile offender	extensive, noncompliant, repeated misconduct and revocations	chronic/severe physical, emotional abuse	chronic/severe physical, emotional, educational, medical neglect	ADHD, IED, Bipolar I, GAD, MDD	Poly substance dependence	pervasive suicidality
B	interference with commerce by robbery	childhood-onset, life-course-persistent, versatile offender	extensive, noncompliant, repeated misconduct and revocations	none	reared in low SES neighborhood	CD, IED, ASPD, ADHD, narcissistic PD	Poly substance dependence	chronic, high homicidal ideation, 25+ violent charges
C	SORNA	childhood-onset, life-course-persistent, versatile offender	refusal to comply with court orders and supervision conditions	physical, emotional, sexual abuse	reared in low SES neighborhoods, transient	MDD	Poly substance abuse	pervasive sexual aggression, high suicidality
D	coercion and enticement	adult-onset, limited arrest history	none prior to instant offense	chronic/severe physical, emotional, sexual abuse	chronic/severe emotional neglect	ADHD, ODD, PTSD, adjustment disorder, borderline PD, PD NOS, GAD, depressive disorder NOS, MDD, ASPD traits, dependent PD traits	alcohol abuse	high suicidality, high self-injurious behaviors; multiple paraphilic disorders
E	armed bank robbery	Childhood-onset, life-course-persistent, versatile offender	Extensive, 10 separate prison commitments	none	none	schizoaffective disorder, MDD, schizophrenia, borderline intellectual functioning	Poly substance dependence	3 murder arrests, command auditory hallucinations, moderate suicidality
F	Possession unregistered firearms	childhood-onset, life-course-persistent, versatile offender	extensive out-of-home placements, three prison commitments	chronic/severe sexual, physical, emotional abuse	chronic/severe physical, emotional, medical neglect; exposure to drugs	MDD, PTSD, dysthymia, IEP, anxiety NOS, pPersonality disorder NOS, avoidant PD, mixed PD w/schizoid and narcissistic traits	Poly substance dependence	pervasive homicidal ideation
G	possession child pornography	adolescent-onset, limited arrest history; extensive lifelong externalizing features	one juvenile and one adult confinement	chronic/severe physical abuse; chronic/severe sexual abuse from multiple perpetrators; prenatal drug exposure	chronic/severe emotional neglect	ADHD, MDD, CD, ODD, GAD, borderline, schizoid, and avoidant PD, dDependent PD traits, pedophilia, sexual dysfunction NOS, fetal alcohol effects, bipolar disorder w/psychotic features, borderline intellectual functioning	none	Multiple paraphilic disorders, fire setting, pervasive suicidality and homicidal ideation, rape fantasies
H	Escape	childhood-onset, life-course-persistent, versatile offender	extensive, noncompliant, repeated misconduct and revocations	chronic physical abuse	physical, emotional, educational, medical neglect	ADHD, pervasive developmental disorder, MDD, bipolar I, ODD, schizoaffective, ASPD	cannabis dependence	pervasive suicidality, self-injurious behaviors, dissociative experiences, thought disturbances
I	Distribution child pornography	adolescent-onset, life-course-persistent, mostly specialized sexual offender	Multiple out-of-home, foster care, juvenile detention, and prison commitments	chronic/severe sexual abuse from multiple perpetrators	physical, emotional neglect	ADHD, bipolar II, PTSD	Poly substance dependence	pervasive suicidality; multiple sexual offenses against children
J	Mailing threatening communication	childhood-onset, life-course-persistent, moderate arrest record	extensive out-of-home placements, six prison commitments, extensive misconduct	physical, emotional abuse	physical, emotional neglect	ADHD, ODD, PTSD, pervasive developmental disorder, bipolar I, dyssomnia, ASPD, MDD, impulse control disorder, reactive attachment disorder, personality disorder NOS	alcohol dependence	pervasive homicidal ideation, highly assaultive across institutional settings
K	Threats against President of the United States	childhood-onset, life-course-persistent, versatile offender	extensive out-of-home placements, five prison commitments, extensive misconduct	sexual abuse	none	schizoaffective, bipolar I, borderline PD, ASPD, PTSD, pedophilia, personality disorder NOS	never used substances	pervasive suicidality and homicidal ideation, extensive self-injurious behaviors,
L	Felon in possession of body armor	childhood-onset, life-course-persistent, versatile offender	extensive out-of-home placements, 12 prison commitments, extensive misconduct	none	none	MDD, ASPD, IED, depressive disorder NOS, adjustment disorder, borderline intellectual functioning	Poly substance dependence	repeatedly violent with law enforcement; pervasive suicidality and homicidal ideation

## Data Availability

Not applicable.
